# Non-antibiotic feed additives production by *Acremonium terricola* solid-fermented *Camellia oleifera* meal

**DOI:** 10.1186/s40643-024-00808-x

**Published:** 2024-09-28

**Authors:** Peng Zhang, Ying Xiong, Luanluan Bi, Haiyan Zhong, Jiali Ren, Bo Zhou

**Affiliations:** 1 Hunan Key Laboratory of Forestry Edible Resources Safety and Processing, Changsha, Hunan 410004 China; 2 Key Laboratory of Cultivation and Protection for Non-Wood Forest Trees of the Ministry of Education, Central South University of Forestry and Technology, Changsha, Hunan 410004 China; 3 Faculty of Food Science and Engineering, Central South University of Forestry and Technology, Changsha, Hunan 410004 China

**Keywords:** Antibiotic-free feed additives, *Acremonium terricola*, *Camellia oleifera* meal, Tea saponin, Cordycepic acid

## Abstract

The *Camellia oleifera* meal (COM), a primary byproduct of oil-tea processing, often being discarded or used as a low-grade fertilizer due to its low value. The underutilization has become a significant bottleneck hindering the high-quality development of the oil-tea industry. In this study, the production of antibiotic-free feed additives through the solid-state fermentation of COM by *Acremonium terricola* was investigated. Our findings revealed that a saponin concentration of 5 mg/mL significantly enhanced the production of cordycepic acid (70.4 mg/g), ergosterol (3.32 mg/g), and chitin (110 mg/g) by *A. terricola*. This concentration also promoted chitin production and the activities of peroxidase (POD) and Na^+^/K^+^-ATPase, thereby maintaining cellular homeostasis and energy balance in *A. terricola*. Solid-state fermented rice bran (RB), wheat bran (WB), and desaponificated COM (containing 2.6 mg/100 g of tea saponin) were all found to be beneficial for increasing the production of cordycepic acid and ergosterol. The blend of COM, RB, and WB in the ratio of 15:65:20 was particularly advantageous for the production and accumulation of cordycepic acid and ergosterol, yielding 1.54 and 1.43 times, 1.27 and 1.37 times, and 1.98 and 5.52 times more than those produced by WB, RB, and COM alone, respectively.Meantime, the difference in contents of sugar and protein in *A. terricola* cultures (ATCs) using combination were not significant compared to RB and WB. These results indicated that COM can partially replace foodstuffs or food by-products to prepare antibiotic-free feed additives by *A. terricola*.

**Graphical Abstract:**

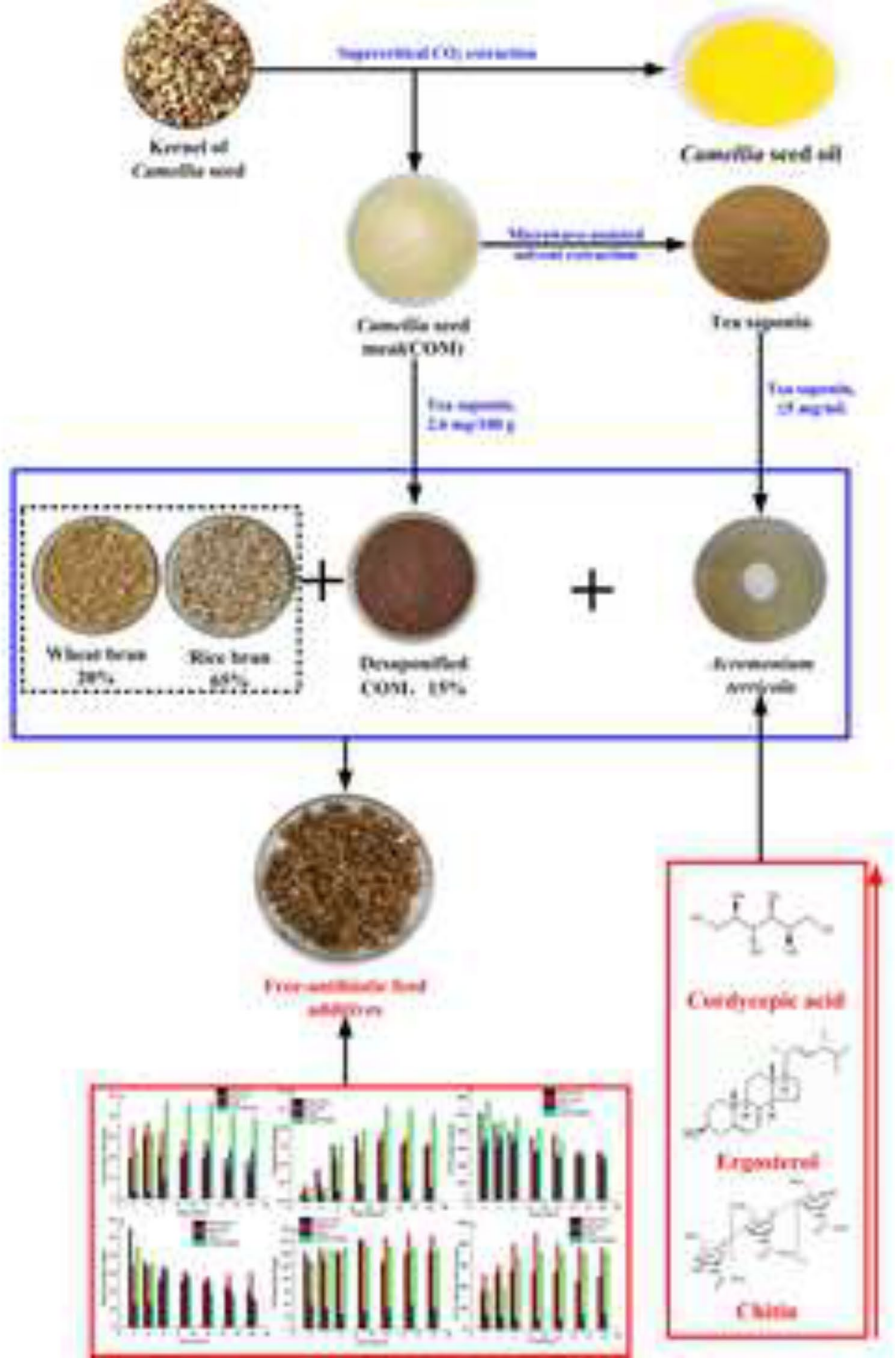

## Introduction

*Acremonium terricola*, a cordyceps-derived fungus isolated from *Cordyceps gurneyiensis* (Fan et al. 1999), is rich in bioactive compounds, including cordycepin, cordycepic acid (D-mannitol), D-mannose, D-galactose, aspartic acid, and glutamic acid (Li et al. 2017), which have diverse biological functions, such as antioxidant, immune regulatory, and anti-inflammatory properties (Li et al. 2018). *A. terricola* cultures (ATCs), generally used as a new type of green feed additive, can play a similar role of antibiotics to promote growth and control diseases for animal (Li et al. 2020), likely increasing the digestibility of feed (Li et al. 2017), laying rate and egg quality of poultry (Xiao et al. 2017), enhancing flavor and nutritional value of milk (Li et al. 2017, 2018), and improving the gastrointestinal flora and immunity of livestock (Li et al. 2016; Zheng et al. 2019; Wang et al. 2019, 2023; Chen et al. 2022). Currently, ATCs was all solid-state fermented from grain (likely corn) and/or grain by-products (likely wheat bran, rice bran, and soybean meal). Therefore, the pursuit of alternative ATCs feedstock sources, especially non-food crops and their by-products enrich in bioactive substrates benefiting for animal growth and development, is vital for maintaining food security and sustainable economic and environmental development.

*Camellia oleifera* Abel is one of the four largest woody oil crops in the world (Luan et al. 2020). *C. oleifera* meal (COM), the residue of camellia seed after oil extraction, contains many bioactive ingredients (including polyphenol (Luan et al. 2020), tea saponin (Yu et al. 2022), protein and polysaccharideglycoside (Li et al. 2010), and is suitable used as a feed substitute after processing through various pretreatments, likely biological fermentation. Nowadays, the utilization of COM was mainly focused on the isolation and purification of tea saponin, as surfactant, used in industry of household cleaning and daily-use chemical (Sharma et al. 2023). Only a small amount of COM was used to clean fish ponds or produce low value-added fertilizer, while most of it was discarded and then resulted in a waste of COM(Yu et al. 2023). Therefore, it is necessary to develop a cost-effective and environment-friendly technology to realize the comprehensive utilization of COM.

Tea saponin, approximately 20% (mg/100 g) of COM, is a natural nonionic surfactant with hydrophilic and lipophilic groups (Sharma et al. 2023) and has many pharmacological effects, likely anti-cancer, anti-inflammatory and antibacterial (Kim et al. 2015). The US Food and Drug Administration has stated that saponin is ‘Generally Recognized as Safe’ for human consumption (Patra and Saxena 2009). More researchers have confirmed that tea saponin could not only inhibit the growth of prokaryotic microorganism (*Escherichia coli* and *Staphylococcus aureus*) (Zhao et al. 2020) and conditional infection fungi (*Candida albicans*) (Yu et al. 2022), but also promote animal growth (Ramdani et al. 2023), modulate rumen fermentation(Hu et al. 2005), impede the release of methane and ammonia and enhance animal immunity(Guajardo-Flores et al. 2013; Chi et al. 2017). However, excess tea saponin also is an anti-nutritional factor, which can affect the palatability of feed and cause acute toxicity for animal, and than significantly inhibit the growth of probiotic organism in animal’s gut (Guajardo-Flores et al. 2013; Chi et al. 2017). Therefore, clarifying the dose-effect relationship between tea saponin concentration and microbial growth is the key to make full use of COM by microbial fermentation.

The purpose of this work is to investigate the mechanism of tea saponin on changes in physiological activity of *A. terricola*, screen the tolerance concentration of tea saponin for *A. terricola*, and provide theoretical basis for *A. terricola* fermented COM.

## Materials and methods

### Materials and strains culture

*Acremonium terricola* (GDMCC 3.680) was purchased from the Guangdong Microbial Culture Collection Center (GDMCC) of China. The composition of seed medium in distilled water was as follows: sugar 30 g/L, peptone 5 g/L, yeast powder 5 g/L, corn starch 10 g/L, KH_2_PO_4_ 1 g/L, MgSO_4_ 0.1 g/L, pH 5–7. The composition of liquid culture medium in distilled water was as follows: sugar 12 g/L, peptone 15 g/L, glucose 35 g/L, yeast powder 15 g/L, corn starch 25 g/L, K_2_HPO_4_ 1.5 g/L, KH_2_PO_4_ 0.5 g/L, MgSO_4_ 0.5 g/L, (NH_4_)_2_SO_4_ 0.05 g/L. All mediums were sterilised at 121℃ for 20 min before use. All medium compositions were obtained from Sinopharm Chemical Reagent Co., Ltd, Shanghai, China.

### Preparation of spore suspensions

After the activated *A. terricola* were cultured on the PDA plate for 7 days at 30^o^C, the spores were scraped off with a sterile cotton swab and then placed into a triangular bottle containing sterile normal saline and glass beads, after shaking in a shaker (ZHWY-2102 C, Zhicheng Analytical Instrument Manufacturing Co., Ltd, Shanghai, China) for 1 h at 30℃, the concentration of spore suspension was adjusted to 10^7^ CFU/mL using handheld automated cell counter (PHCC360KIT/Scepter 3.0, Merck Millipore, Germany).

### Solid-state culture

The composition of solid-state culture medium was as follows: 20 g of COM or rice bran (RB) or wheat bran (WB), 12 mL of water (approximately 70% water content), natural pH, sterilized at 121℃ for 20 min. 0.3 mL of spores suspension (10^7^ CFU/mL) of *A. terricola* cultured on PDA agar slants was transferred into 250 ml of shake flasks containing 20 g of solid-state culture medium, and then incubated at 30℃ and agitated every 48 h to increase aeration. RB and WB were free provided by Hunan Jinjian Rice Co.Ltd. COM, which is the residue of oil production by supercritical carbon dioxide extraction from seed of *Camellia oleifera* Abel, was provided by Hunan Health-Guard Bio-Tech Inc., Yongzhou, China.

### Antibacterial activity of tea saponin on *A. terricola*

The colony diameter was measured as following: 10 µL spore suspension (10^7^ CFU/mL) drops were added to the center of PDA medium containing different concentration tea saponin (0 mg/mL, 2 mg/mL, 4 mg/mL, 6 mg/mL, 8 mg/mL, 10 mg/mL, and 12 mg/mL)(Solarbio Science and Technology Co., Ltd, Beijing, China). The PDA plate was cultured at 30℃ for 7 days, and the diameter (mm) of colony zone was measured with a calliper according to the method (Wei et al. 2020).

The inhibitory zone diameter was measured by the punching method as following: 100 µL spore suspension (10^7^ CFU/mL) was uniformly coated on PDA plate, and then three evenly spaced round holes has been punched with a hole punch (diameter of 6 mm) on PDA plate. 100 µL of tea saponin solutions with different concentration (30 mg/mL, 15 mg/mL, 10 mg/mL, 5 mg/mL, 2 mg/mL, 0 mg/mL) was added into hole. After culturing for 48 h at 30℃, and the diameter (mm) of inhibitory zone was measured with a calliper according to the method (Wei et al. 2020).

### Minimum inhibitory concentration (MIC) and minimum bactericidal concentration (MBC) of tea saponin on *A. terricola*

MIC and MBC experiments were performed using the double dilution method (Zhao et al. 2020) as following:100µL spore suspension (10^7^ CFU/mL) was placed into a sterile test tube containing 5 mL of tea saponin solution with different concentration (30 mg/mL, 15 mg/mL, 10 mg/mL, 5 mg/mL, 2 mg/mL). After incubation at 150 r/min and 30℃ in a shaker (ZHWY-2102 C, Zhicheng Analytical Instrument Manufacturing Co., Ltd, Shanghai, China) for 24 h, 100 µL culture was uniformly coated on PDA plate and continually inoculated at 30℃ for 7 days. The concentration of tea saponin corresponding to the start of inhibition zone was the MIC, and the lowest concentration of tea saponin without *A. terricola* growth was the MBC.

### Microscope analysis of *A. terricola*

Mycelium of *A. terricola* grown (30℃/7 d) on PDA plate was collected to observe using optical microscope (BM1000, Nanjing Jiangnan Yongxin Optical Co., Ltd., China).

For scanning electron microscopy (SEM), the collected *A. terricola* from PDA plate was immobilized at 4℃ for 6 h by 2.5% glutaraldehyde and washed three times with phosphate buffer solution (PBS) (0.2 M, pH 7.0) for 15 min, and then dehydrated by a series of 10 ~ 100% ethanol for 15 min (twice). After drying at room temperature (25℃) and spraying with gold, samples were used for SEM observation (Hitachi SU8010 SEM, working voltage: 80 kV, amplification factor: 80,000).

For transmission electron microscope (TEM), the collected *A. terricola* from PDA plate was washed three times using sterile water, and fixed for 4 h with 1 mL of fixative containing 3% paraformaldehyde and 4% glutaraldehyde in darkness. Samples were washed three times with PBS (0.2 M, pH7.0), and then dehydrated with different concentrations of ethanol solution (30%, 50%, 70%, 85%, 95%, and 100%). After embedding in resin and further polymerisation at 65℃ for 2 days (Li et al. 2016), samples were observed using TEM (Hitachi H-7650 TEM, working voltage: 60 kV, amplification factor: 3,000).

### Analysis of integrity and permeability of wall and membrane in *A. terricola*

The cell wall integrity of *A. terricola* was analyzed with a calcofluor white (CFW) probe as following: *A. terricola* growth (150 r/min, 30^o^C, 7 d) in submerged culture medium was collected by centrifuging (Sorvall™ LYNX 6000, Thermo Scientific™, USA) at 8000 g for 10 min, the precipitate was washed three times using PBS (0.2 M, pH 7.0) and then suspended in 100 ml of PBS buffer (0.2 M, pH 7.0). 3 mL suspension with a CFW probe (2.5 µg/mL) was used to determine cell wall integrity through fluorescence intensity of supernatant using G-98,000 A fluorospectrophotometer (Agilent, USA) with 368 nm of excitation and 442 nm of emission.

The integrity/outer membrane permeability of cell membrane in *A. terricola* was analyzed with probe of propidium iodide (PI) (Riccardi and Nicoletti 2006) / 1-nitrogen-benzenaphthaline (NPN) (Helander and Mattila-Sandholm 2000). The cell integrity/outer membrane permeability was determined by fluorescence intensity of supernatant using G-98,000 A fluorospectrophotometer (Agilent, USA) with 535/338 nm of excitation and 630/417 nm of emission, respectively.

The permeability of cytomembrane in *A. terricola* was expressed by relative conductivity (RC) as following: *A. terricola* cultured (150 r/min, 30^o^C, 7 d) in submerged culture medium was collected by centrifuging (Sorvall™ LYNX 6000, Thermo Scientific™, USA) at 8000 g for 10 min, the precipitate was washed three times using ultra-pure water and then suspended in 20 mL of ultra-pure water elution for 24 h. The supernatant (8000 g / 10 min) was used to measure extracellular conductivity (extr-C) using conductivity meter (Hongyi DDS307, Shanghai). The precipitation was again placed into 20 mL of ultra-pure water and then boiled 30 min in water bath, the supernatant (8000 g / 10 min) was used to measure intracellular conductivity (intr-C) using conductivity meter (Hongyi DDS307, Shanghai). RC(%)=(extr-C)/((extr-C)+(intr-C))$\:\times\:$100.

### Determination of ROS content and enzymes activity in *A. terricola* intracellular extracts

The intracellular extracts from *A. terricola* was prepared as following described. After 7 days cultivation, the fermentation broth was centrifuged (Sorvall™ LYNX 6000, Thermo Scientific™, USA) at 10,000 g for 5 min, the collected mycelia was washed 3 times with phosphate buffer (pH 7.0, 50 mmol/L), and then freeze-dried (-48℃/4 Pa) (FreeZone 6 –84 C Console Freeze Dryers, Labconco Corporation, USA) and stored at 4 ℃ until for use. The lyophilized mycelia was resuspend in phosphate buffer (pH 7.0, 50 mmol/L) and then fully disrupted by sonication (Scientz IID, Ningbo Scientz Biotechnology Co., Ltd, China) as following condition: working 2 s, intermittent 3 s, full time 25 min, power 400 W, protection temperature 4℃. The mycelia disruption solution was centrifuged at 4℃, 4,500 g for 15 min. The collected supernatant was *A. terricola* intracellular extracts and stored at 4 ℃ until for use.

Content of reduce reactive oxygen species (ROS) and ATP, and activity of enzymes (including superoxide dismutase (SOD), catalase (CAT), peroxidase (POD) and glutathione peroxidase (GSH-PX), ATPase and electron transport chain Complex I, II, III, IV, ) in intracellular extracts from *A. terricola* were all assayed using the assay kit (manufactured by Nanjing Jiancheng Bioengineering Institute, China) according to the manufacturer’s instructions.

### Determination of cordycepic acid, ergosterol and chitin in *A. terricola*

The lyophilized mycelia (0.2 g) were grinded in a mortar (0.2 g quartz sand) for 10 min using absolute methanol, the finely ground homogenate was transferred into centrifuge tube (100 mL), then absolute methanol (40 mL) was added and placed in an ultrasonic cleaner (SBL-10DT, Ningbo Scientz Biotechnology Co., Ltd, China) for 10 min as following condition: power 150 W, frequency 40 kHz. After centrifugation (Sorvall™ LYNX 6000, Thermo Scientific™, USA) at 8000 g for 10 min, the supernatant was filled with anhydrous methanol to 50 mL and then filtered using microporous organic phase filter membrane (0.45 μm) for use.

Content of cordycepic acid (Lin et al. 2016), chitin (Costa-de‐Oliveira et al. 2013) and ergosterol (Liu et al. 2022) in *A. terricola* was assayed and expressed with mg/g, respectively. The tea saponin content was determined according to the report (Le Bot et al. 2022). Cordycepic acid, ergosterol and chitin standard compounds (Sigma, USA) were used to prepare standard curves.

### Data analysis

All the data showed in tables and figures were expressed as mean ± standard deviation of the three replicates. All figures were drawn with OriginPro 8.0 (OriginLab Corporation, Northampton, MA 01060, USA) and Microsoft Office Visio 2003 (Microsoft Corporation, Redmond, WA 98052, USA).

## Results

### MIC and MBC of tea saponin against *A. terricola*

**Fig. 1:**
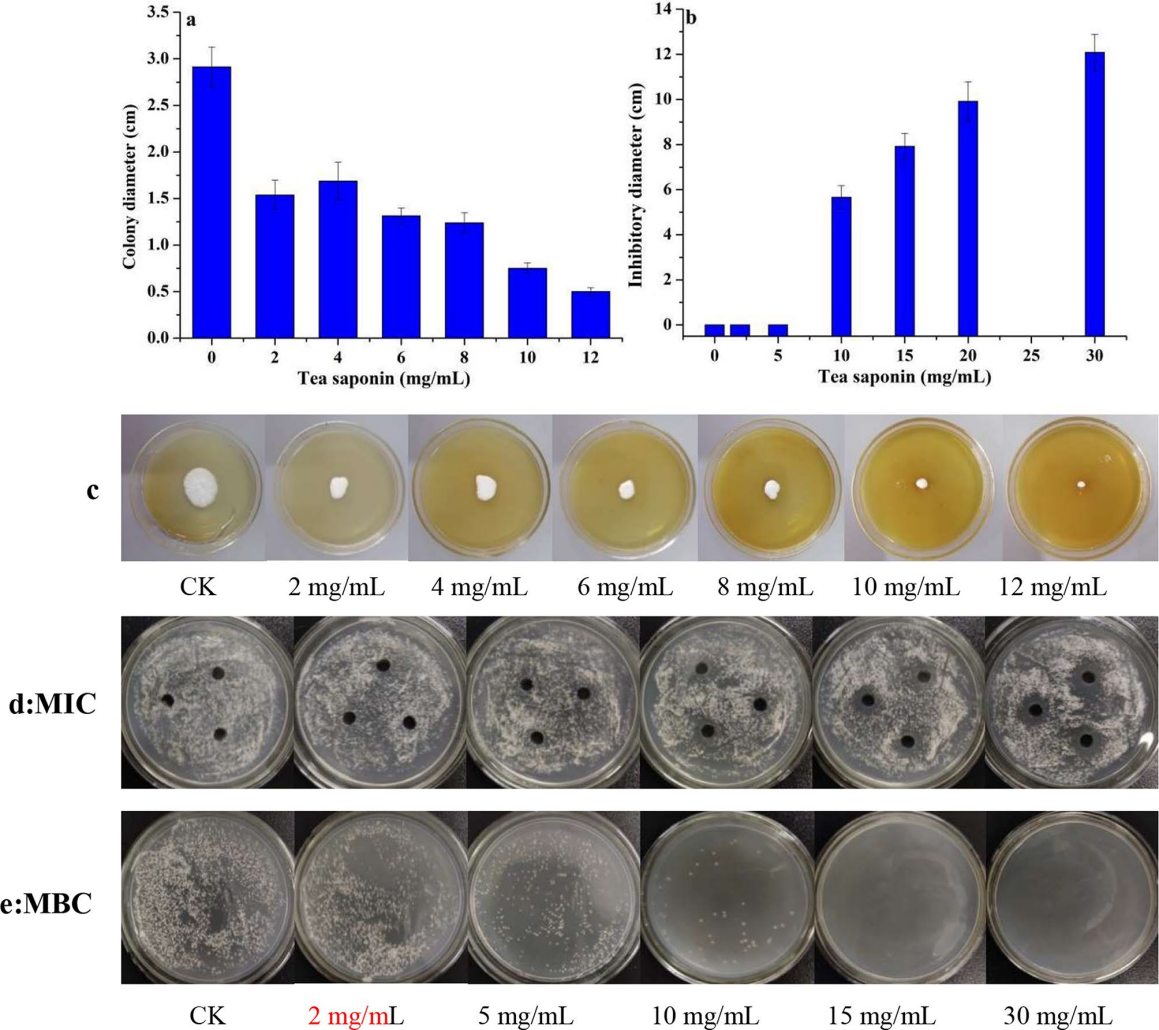
Effect of tea saponin on *A. terricola* growth on PDA plate. **a** and **c**: Colony diameter; **b**: Inhibitory diameter; **d**: MIC; **e**: MBC. *Notes* CK represents without tea saponin addition

The effect of tea saponin against *A. terricola* was proportional to its concentration (Fig. 1a and c). When the concentration of tea saponin was 2 mg/mL, 8 mg/mL and 12 mg/mL, the colony diameter decreased from 2.91 cm to 1.54 cm, 1.24 cm and 0.5 cm, and the inhibition rate reached 47.1%, 19.5% and 82.8%, respectively. Interestingly, the colony diameter of *A. terricola* only decreased from 1.54 cm to 1.24 cm in range from 2 mg/mL to 8 mg/mL of tea saponin, and which showed that the protection mechanisms in *A. terricola* has been induced.

The concentration of tea saponin against *A. terricola* with significant difference in antibacterial zone was not less than or equal to 5 mg/mL (Fig. 1b) but greater than or equal to 10 mg/mL (*p* < 0.05) (Fig. 1d). After culturing 7 days, the colony of *A. terricola* did not appear on PDA plates containing tea saponin (15 mg/mL) (Fig. 1e). The results showed that the MIC / MBC of tea saponin against *A. terricola* was 10 / 15 mg/mL in this work.

All above results indicated tea saponin had certain antibacterial effect on *A. terricola*, and the MIC and MBC of tea saponin against *A. terricola* was 10 mg/mL and 15 mg/mL, respectively.

### Mycelial and spore morphology changes in *A. terricola* tolorance to tea saponin

**Fig. 2:**
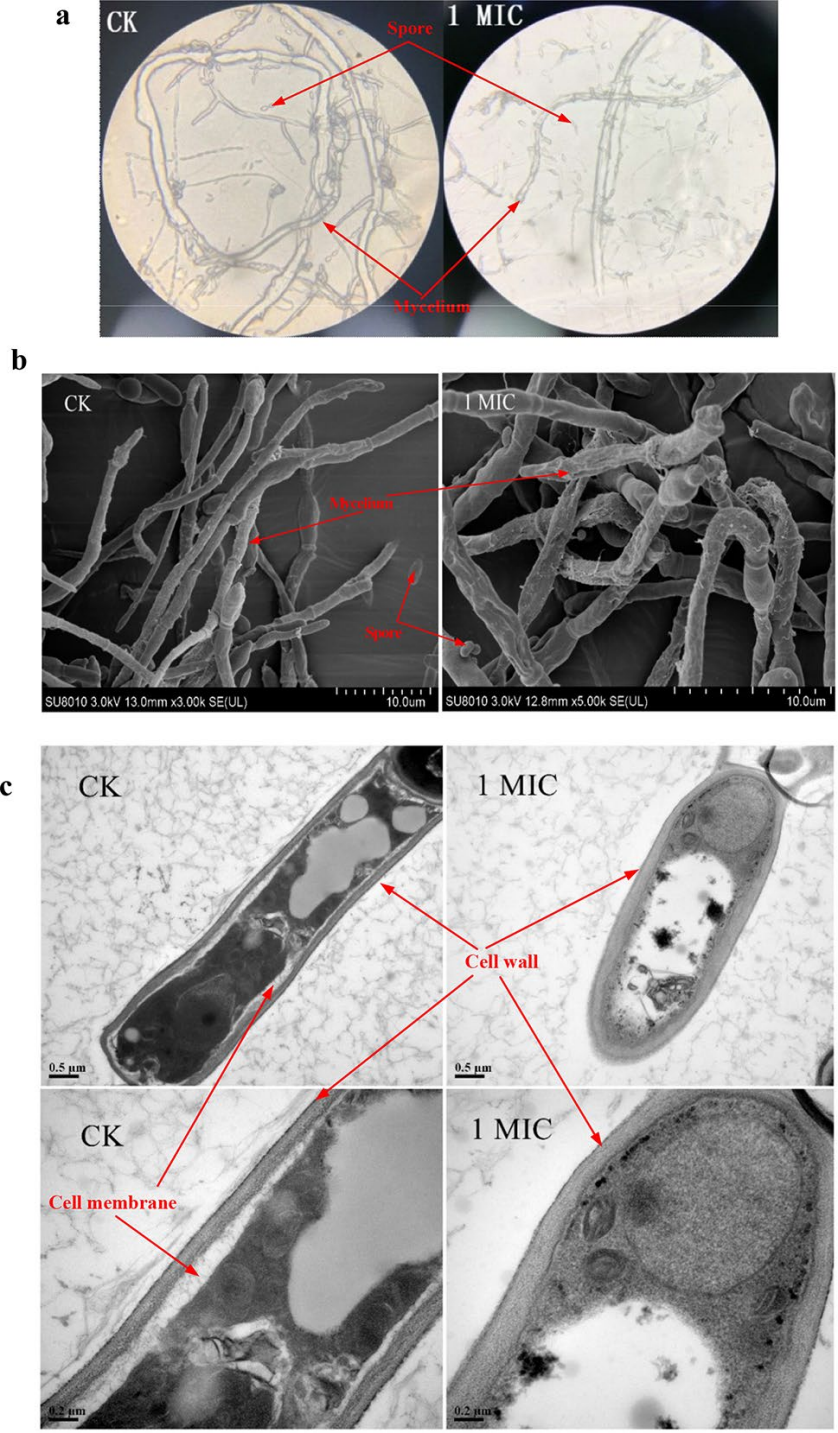
Mycelial morphology changes of *A. terricola*. **a**: Mycelium morphology of *A. terricola* observed by optical microscope; **b**: Mycelium morphology of *A. terricola* observed by SEM; **c**: Mycelium morphology of *A. terricola* observed by TEM (0.5 μm, 0.2 μm). *Notes* CK represents without tea saponin addition; 1MIC represents 10 mg/mL of tea saponin addition

The mycelium of *A. terricola* growth on PDA without tea saponin addition was long, smooth and robust, uniform in thickness, and the spore was numerous and arranged tightly and regularly observed by optical microscope (Fig. 2a). However, the mycelium of *A. terricola* growth on PDA with tea saponin (10 mg/mL) was deformed, twisted, and obviously wrinkled, the thickness was not uniform, and the spores were few and scattered (Fig. 2a). The results from SEM observation showed that the mycelium of *A. terricola* has serious distortion, obvious shrinkage, rough surface and abnormal morphology, and which was consistent with those observed by optical microscope (Fig. 2b). The results of TEM showed that the cell wall and membrane of mycelium in *A. terricola*, grown on PDA without tea saponin, was complete and clear, the cytoplasm and organelle was uniform and clear, respectively (Fig. 2c). Nevertheless, the cell wall of mycelium in *A. terricola* growth on PDA with tea saponin(10 mg/mL), was loose, blurring for cytomembrane of mycelium, leakage of vacuole contents, and the internal organelle structure was significantly damaged. These results showed that tea saponin used in present work had adverse effects on mycelium morphology of *A. terricola.*

**Fig. 3:**
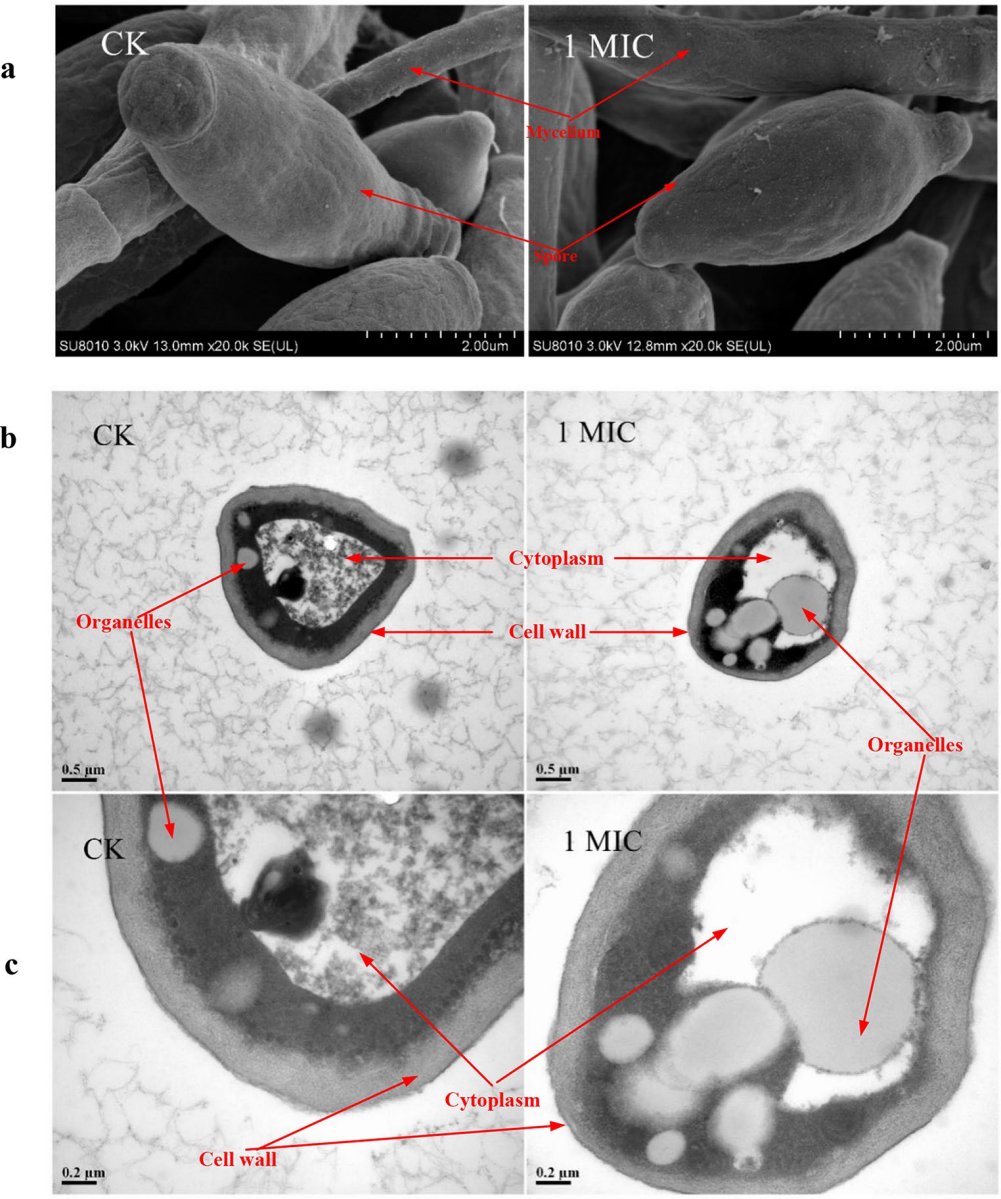
Spore morphology changes of *A. terricola*. **a**: Spore morphology of *A. terricola* observed by SEM (2 μm); **b**: Spore morphology of *A. terricola* observed by TEM (0.5 μm, 0.2 μm). *Notes* CK represents without tea saponin addition; 1MIC represents 10 mg/mL of tea saponin addition

The spores of *A. terricola* growth on PDA without tea saponin addition were numerous, and arranged tightly and regularly observed by optical microscope (Fig. 3a). The cell wall and membrane were tightly fitted, the boundary was clear, and the vacuole was complete from SEM observation (Fig. 3b and c). However, tea saponin (10 mg/mL) had a significant influence on the external morphology of spore from SEM observation (Fig. 3a). These results about TEM also further confirmed that tea saponin caused changes in spore morphology of *A. terricola*, cell wall has irregular edges and loose structure, blurring for cytomembrane of mycelium, leakage of vacuole contents, and the internal organelle structure was significantly damaged (Fig. 3b and c). These results showed that tea saponin used in present work had adverse effects on spore morphology of *A. terricola.*

All above results indicated that tea saponin changed the normal morphology of mycelium and spore, and then affected the growth and biological activity of *A. terricola.*

### Integrity changes in cell wall and cytomembrane of *A. terricola* tolorance to tea saponin

**Fig. 4:**
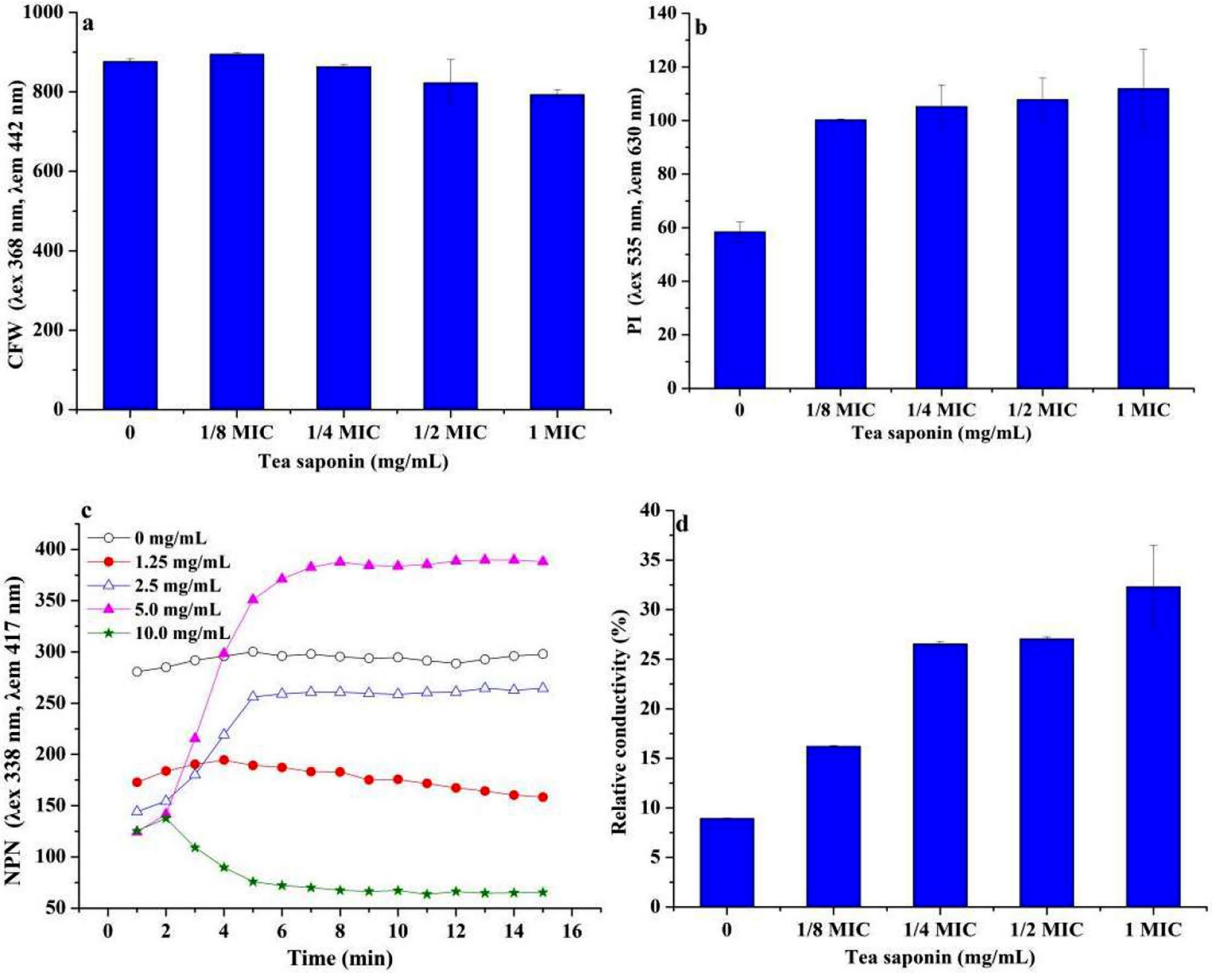
Effect of tea saponin on cell homeostasis in *A. terricola*. **a**: cell wall integrity; **b**: cell membrane integrity; **c**: cell membrane permeability; **d**: Relative conductivity. *Notes* MIC represent with 10 mg/mL of tea saponin. MIC represents 10 mg/mL of tea saponin

CFW fluorescence value in *A. terricola* decreased slightly from 876 (0 mg/mL of tea saponin addition) to 823 (5 mg/mL of tea saponin addition) (Fig. 4a). CFW fluorescence values for tea saponin addition (10 mg/mL) decreased by only 9.44% (*p* ≤ 0.05) compared to the control (Fig. 4a). This result showed there has no obvious influence of tea saponin on cell wall integrity of *A. terricola* in present work.

The PI fluorescence value in *A. terricola* cultured without tea saponin addition was 58.4 (Fig. 4b). The PI fluorescence value was 100, 105, 108 and 112 for 1.25 mg/mL, 2.5 mg/mL, 5 mg/mL and 10 mg/mL of tea saponin addition (Fig. 4b), respectively. The PI fluorescence value was increased by 71.6%, 80.1%, 84.6% and 91.5% (*p* ≤ 0.05)(Fig. 4b), respectively. This result showed that tea saponin had an adverse influence on the integrity of cell membrane, and the concentration of tea saponin (1.25 mg/mL ~ 10 mg/mL) was tolerance concentration for *A. terricola*.

Obvious different change in NPN fluorescence values indicated that tea saponin significantly altered cell membrane integrity of *A. terricola* (Fig. 4c). Compared without tea saponin addition, the tea saponin of 1.25 mg/mL, 2.5 mg/mL, 5 mg/mL and 10 mg/mL resulted in a decrease in NPN values of *A. terricola* with 38.4%, 48.6%, 55.7% and 55.3% (Fig. 4c), respectively. It was very interesting that the NPN fluorescence value showed a decreasing trend for 10 mg/mL of tea saponin addition, the opposite was true for 2.5 mg/mL and 5 mg/mL of tea saponin additions (Fig. 4c). For example, the NPN fluorescence value at 10 min was increased by 79.5% and 208% than that at 1 min (Fig. 4c). Whereas the difference in NPN fluorescence value for tea saponin addition(1.25 mg/mL) was insignificant, likely, the NPN fluorescence value at 15 min was 158 and it was 91.6 times higher than that at 1 min (Fig. 4c). The relative electrical conductivity of *A. terricola* cultured with tea saponin addition(1.25 mg/mL, 2.5 mg/mL, 5 mg/mL and 10 mg/mL, respectively) was 16.2%, 26.5%, 27.1% and 32.3%, and it was 1.81, 2.97, 3.03, 3.62 times than that of without tea saponin addition (Fig. 4d), respectively. These results further indicated that tea saponin significantly increased the permeability of cell membrane of *A. terricola*. It was also interesting that the relative conductivities difference between 2.5 mg/mL and 5 mg/mL of tea saponin addition was not significant (Fig. 4d), this result indicated that tea saponin (2.5 mg/mL ~ 5 mg/mL) induced the generation of protective mechanisms in *A. terricola* to reduce the cell membrane permeability and thus maintain membrane homeostasis.

### Changes in antioxidant enzyme activity and reactive oxygen content of *A. terricola* tolorance to tea saponin

**Fig. 5:**
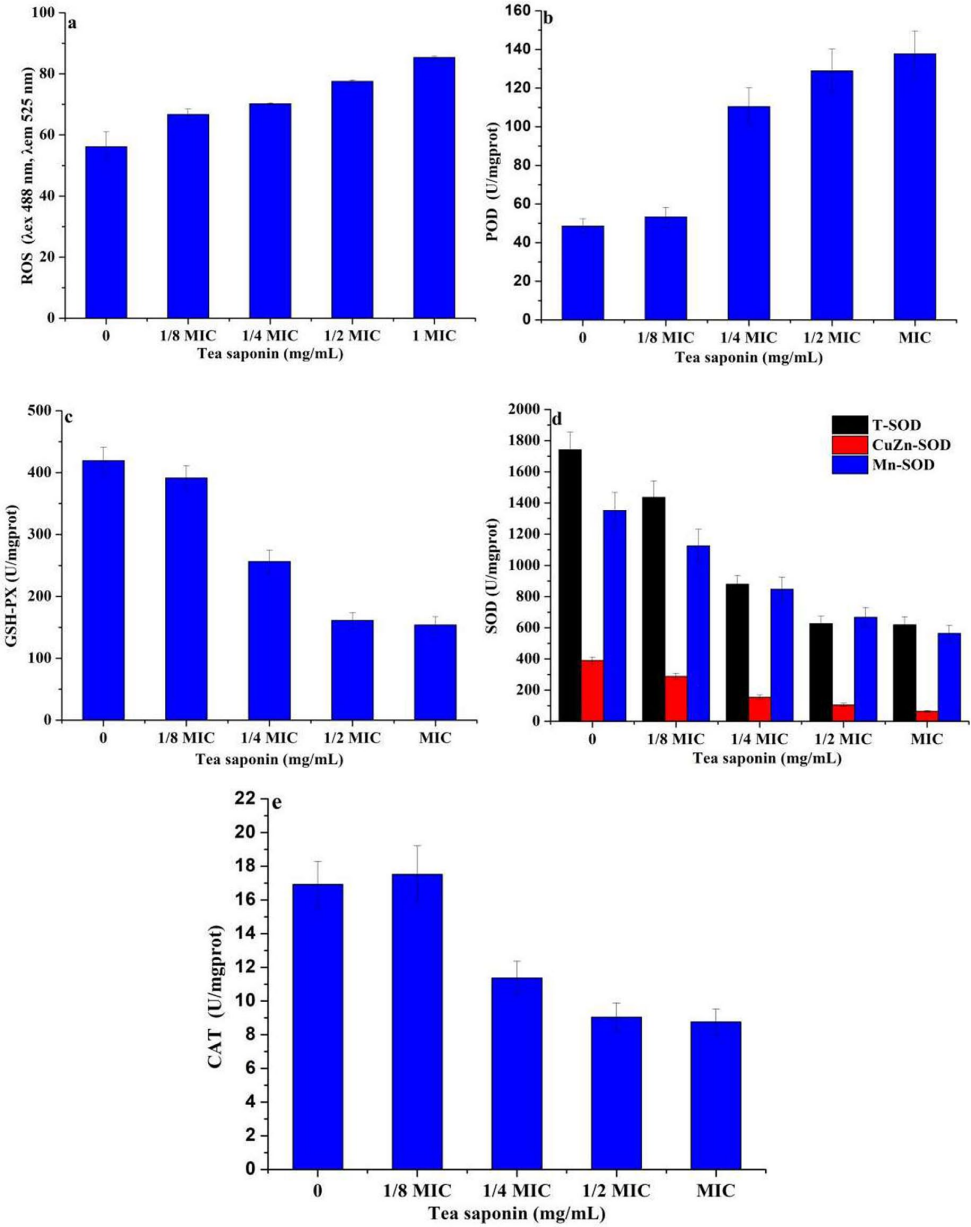
Effect of tea saponin on intracellular antioxidant enzymes activity in *A. terricola*. **a**: ROS content; **b**: POD; **c**: GSH-PX; **d**: SOD; **e**: CAT. *Notes* MIC represents 10 mg/mL of tea saponin

The ROS content in *A. terricola* cultured with tea saponin (1.25 mg/mL, 2.5 mg/mL, 5 mg/mL and 10 mg/mL, respectively) was 1.19 times, 1.25 times, 1.38 times and 1.52 times than that of without tea saponin addition (Fig. 5a), respectively. These results indicated tea saponin could enhance the accumulation of ROS in *A. terricola*, meanwhile, *A. terricola* may produce related substances to reduce ROS accumulation or lower the rate of their generation.

As the concentration of tea saponin increased, the activities of all enzymes showed a decreasing trend except for POD. Notably, the difference in activity of POD (Fig. 5b), GSH-PX (Fig. 5c) and CAT (Fig. 5e) between 0 mg/mL and 1.25 mg/mL of tea saponin addition was not obvious, the opposite was true for SOD (Fig. 5d). These results indicated that the increase in ROS content may be caused by the decrease in SOD activity in *A. terricola* under 1.25 mg/mL of tea saponin addition. In terms of *A. terricola* cultured with 10 mg/mL of tea saponin addition, the activity of CAT (Fig. 5e) and GSH-PX (Fig. 5c) was decreased by 50% and 58.8%, respectively, while the activity of POD increased by 183% (Fig. 5b). The activities of T-SOD, CuZn-SOD and Mn-SOD was decreased by 64.0%, 83.7% and 58.3% (Fig. 5d), respectively.

All above results indicated tea saponin promoted the accumulation of ROS in *A. terricola*, and the detrimental effects of ROS on *A. terricola* will be eliminated by increasing POD activity.

### Changes in mitochondrial complex enzyme activity of *A. terricola* tolorance to tea saponin

**Fig. 6:**
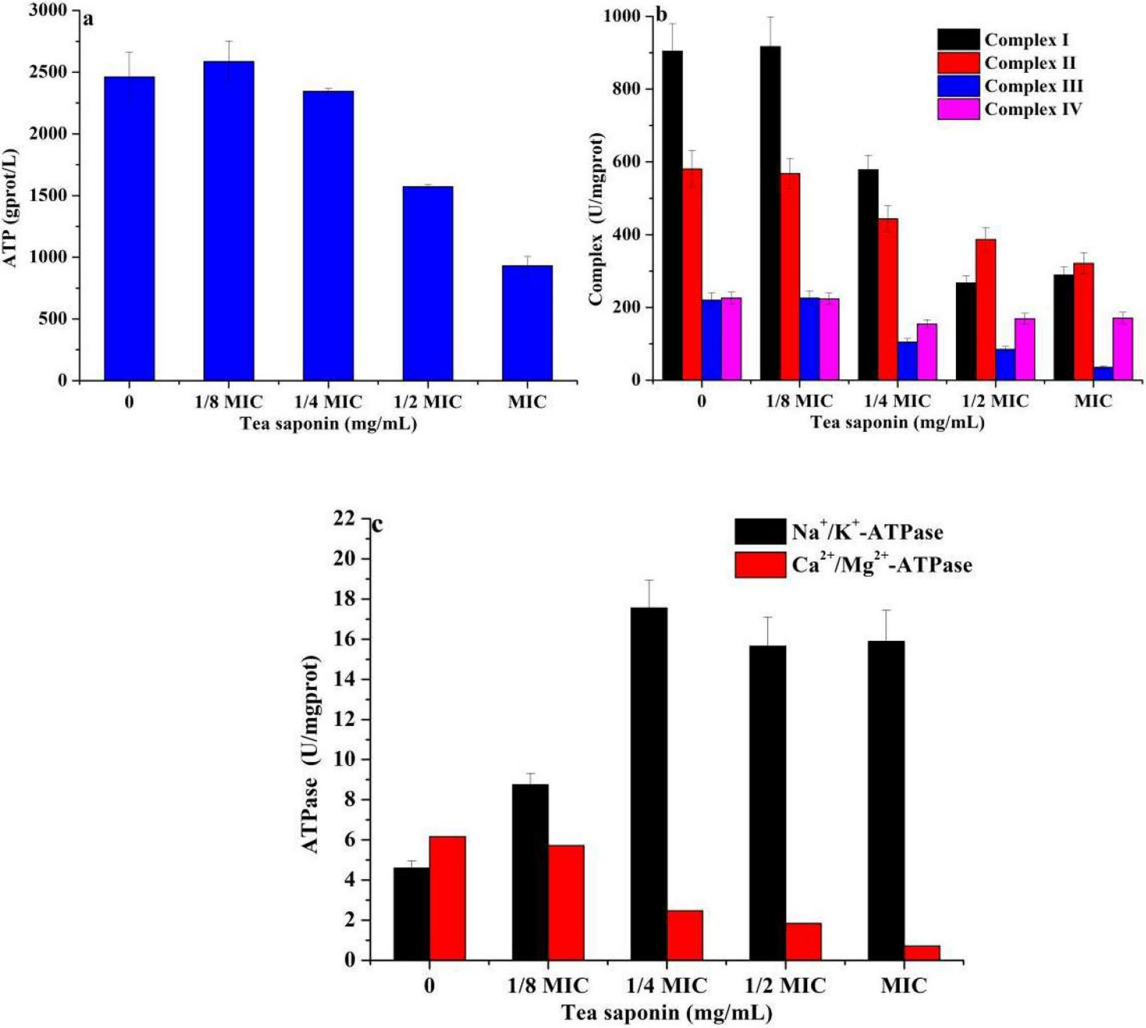
Effect of tea saponin on respiratory chain of *A. terricola.***a**: ATP content ; **b**: Mitochondrial complex enzyme activities; **c**: Enzyme activity of ATPase. *Notes* MIC represents 10 mg/mL of tea saponin

Compared without tea saponin addition, 10 mg/mL of tea saponin resulted in a decrease of 62.1% in ATP content in *A. terricola*, however, the difference in ATP production by *A. terricola* cultured with tea saponin addition (from 1.25 mg/mL to 2.5 mg/mL) was not significant (Fig. 6a). Meantime, 10 mg/mL of tea saponin addition caused the decrease in activity of mitochondrial complex I, II, III and IV with 67.8%, 42.2%, 83.5% and 24.4% (Fig. 6b), respectively. The activity of Ca^2+^/Mg^2+^-ATPase and Na^+^/K^+^-ATPase was decreased by 86.9% and increased by 251% (Fig. 6c), respectively. Interestingly, the difference in activity of mitochondrial complex (I, II, III and IV) (Fig. 6b) and Ca^2+^/Mg^2+^-ATPase in *A. terricola* cultured with tea saponin addition (from 0 mg/mL to 1.25 mg/mL) was not obvious. These results indicated that tea saponin inhibited the activity of mitochondrial mitochondrial complex (I, II, III and IV) and Ca^2+^/Mg^2+^-ATPase, and increased the Na^+^/K^+^-ATPase activity, and then ultimately lead to a decrease in ATP content.

### Changes in content of cordycepic acid, ergosterol and chitin producing by A. terricola liquid cultured against tea saponin

**Fig. 7:**
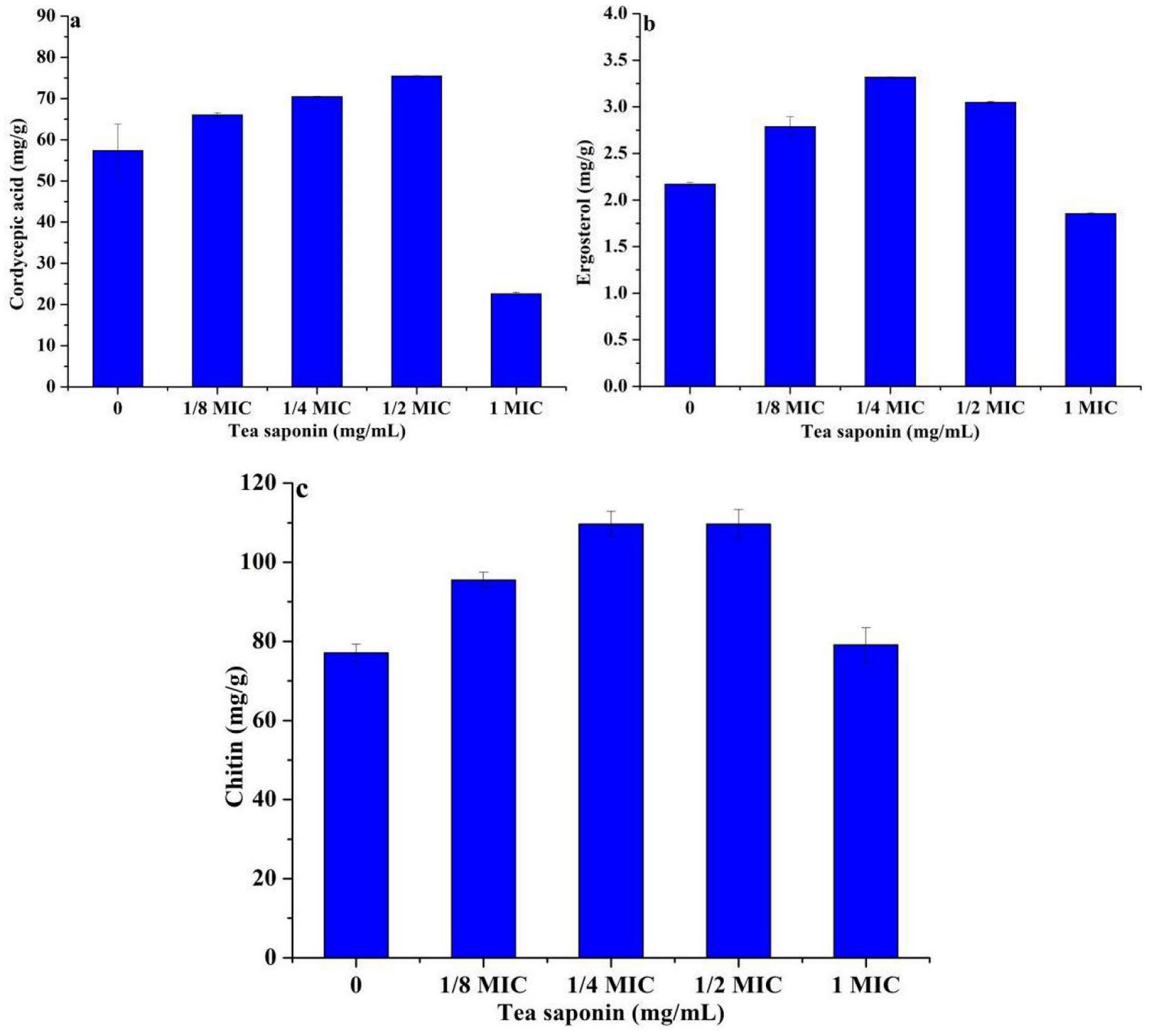
Effect of tea saponin on production of cordycepic acid (**a**), ergosterol (**b**) and chitin (**c**) from *A. terricola. Notes* MIC represents 10 mg/mL of tea saponin

In present work, compared without tea saponin addition, the cordycepic acid content in *A. terricola*, which was liquid cultured with different tea saponin addition (1.25 mg/mL, 2.5 mg/mL, 5 mg/mL and 10 mg/mL), has been increased by 15.1%, 23.7%, 31.6% and − 60.6% (Fig. 7a), respectively. These results indicated tea saponin (≤ 5.0 mg/mL) could significantly promote cordycepic acid production by *A. terricola.*

In case of ergosterol production by *A. terricola* against tea saponin, the highest ergosterol content in *A. terricola* liquid cultured with tea saponin addition (2.5 mg/mL) was 3.32 mg/g and 52.3% higher than that of without tea saponin addition (Fig. 7b), however, the ergosterol content in *A. terricola* liquid cultured with 10 mg/mL of tea saponin addition has been decreased by 14.7% (Fig. 7b). These results indicated that tea saponin (≤ 5 mg/mL) could enhance the ergosterol production by *A. terricola.*

Compared without tea saponin addition, the chitin content in *A. terricola*, which was liquid cultured with different tea saponin addition (1.25 mg/mL, 2.5 mg/mL, 5.0 mg/mL and 10.0 mg/mL), has been increased by 29.1%, 42.2%, 42.2% and 2.57% (Fig. 7c), respectively. The highest content (110 mg/g) of chitin was found at 1.25 mg/mL of tea saponin addition, which was increased by 42.2% compared without tea saponin addition. Interestingly, the difference in chitin content was very unobvious between 10 mg/mL and 0 mg/mL of tea saponin addition (*p* ≤ 0.05), which was 79.9 mg/g and 77.1 mg/g, respectively. These results indicated that tea saponin could improve the chitin production by *A. terricola* in this work.

All above results indicated that tea saponin (≤ 5 mg/mL) could increase the production of cordycepic acid, ergosterol and chitin in *A. terricola.*

### Changes in content of cordycepic acid and ergosterol from ATCs solid-state fermentation using COM, RB and WB

According to the report (He et al. 2014), microwave-assisted extraction has been used to extract tea saponin from COM and the content of tea saponin in COM decreased from 21.2% (mg/100 g) to 2.6% (mg/100 g) in this work.

**Fig. 8:**
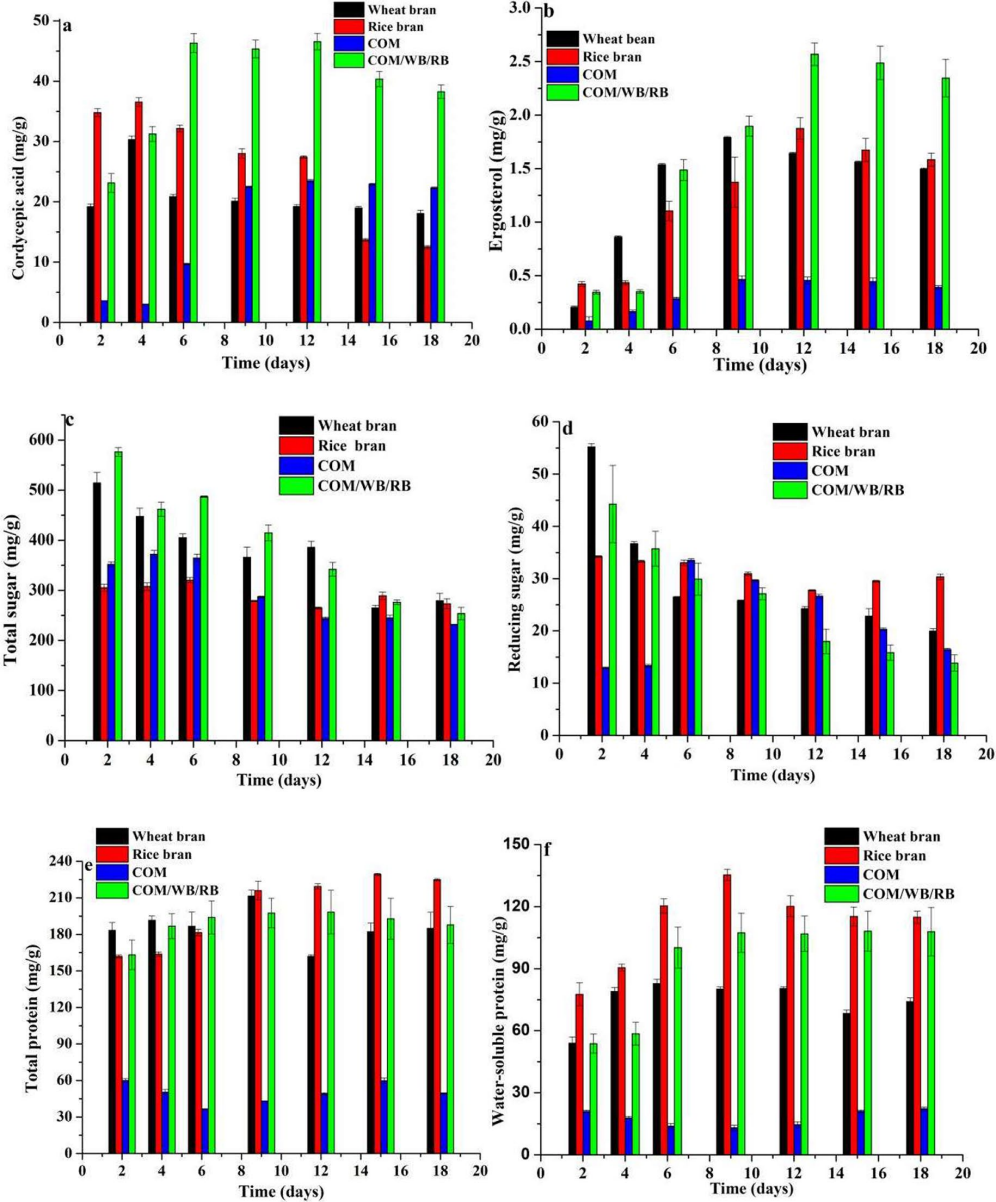
Changes of nutrient contents in *A. terricola* cultures by solid-state fermentation using COM, wheat bran, rice bran and complex (COM: RB: WB = 15:65:20). **a**: Cordycepic acid; **b**: Ergosterol; **c**: Total sugar; **d**: Reducing sugar; **e**: Total protein; **f**: Water-soluble protein

In terms of cordycepic acid, RB and WB were more beneficial to the production of cordycepic acid than COM, especially in the early fermentation stage (Fig. 8a). The maxium content of cordycepic acid reached on the 4th day of solid-fermentation using WB (30.3 mg/g) and RB (36.5 mg/g), then its content decreased to 18.1 mg/g and 12.5 mg/g on the 18th day (Fig. 8a), respectively. When COM was used as the substrate for solid-fermentation, the content of cordycepic acid was reached the maximum (23.5 mg/g) only after 12 days of solid-fermentation and then remained with 22.3 mg/g on 18th day. These results demonstrated that RB and WB were all favorable for cordycepic acid production in the not late stage but early stage of solid-state fermentation, the opposite was true for COM (Fig. 8a). When combination (COM: RB: WB = 15:65:20) was used as substrate for *A. terricola* fermentation, the content of cordycepic acid in ATCs reached 23.1 mg/g, 46.3 mg/g, 46.6 mg/g and 38.3 mg/g on the 2nd, 6th, 12th and 18th day of solid-fermentation (Fig. 8a), respectively, and its maximum content was 1.54, 1.27, and 1.98 times than that of WB, RB, and COM (*p* ≤ 0.05), respectively. These results indicated that combination (COM: RB: WB = 15:65:20) was favorable for cordycepic acid production and accumulation.

As far as ergosterol was concerned, RB and WB were more beneficial to the production of ergosterol than COM (Fig. 8b). When WB, RB and COM was used as substrate for solid-fermentation, the maximum content of ergosterol reached with 1.79 mg/g, 1.88 mg/g and 0.47 mg/g on the 9th, 12th and 9th day of solid-fermentation (Fig. 8b), respectively, and its accumulation did not change significantly with the extension of fermentation time. These results indicated that WB and RB were more favorable to ergosterol production than COM. When combination (COM: RB: WB = 15:65:20) was used as substrate for solid-fermentation, the content of ergosterol reached with 0.35 mg/g, 1.49 mg/g, 2.57 mg/g and 2.35 mg/g on the 2nd, 6th, 12th and 18th day of solid-fermentation, respectively, and its maximum content was 1.43, 1.37, and 5.52 times than that of WB, RB, and COM (*p* ≤ 0.05), respectively. These results indicated that combination (COM: RB: WB = 15:65:20) was favorable for ergosterol production and accumulation.

## Discussion

### MIC and MBC of tea saponin against *A. terricola*

Currently, many studies have proven that tea saponin has many biological effects, such as antioxidant (Li et al. 2014), anti-tumour (Zhao et al. 2015), antibacterial(Yu et al. 2022), hypoglycemic (Di et al. 2017), and anti-inflammatory (Yang et al. 2014). Therefore, high-value utilization of COM was mainly focused on the extraction and application of tea saponin, and the applications for tea saponin extracted from COM was mainly focused in the fields of medicine, daily chemical and biopesticide based on its excellent bacteriostatic and bactericidal properties.

Theoretically, the concentration of a substance that causes the zone of inhibition to just appear and completely inhibit for cell growth is the MIC and MBC of that substance (Menezes et al. 2022), respectively. In present work, the concentration of tea saponin (2 mg/mL ~ 8 mg/mL) could induce *A. terricola* to produce positive stress protection mechanism (Fig. 1). The MIC and MBC of tea saponin against *A. terricola* was 10 mg/mL and 15 mg/mL (Fig. 1), respectively, and which was much higher than that of *Candida albicans* (0.78 mg/mL and 3.12 mg/mL) and *Saccharomyces cerevisiae* (1.56 mg/mL and 6.25 mg/mL) (Yu et al. 2022). The results indicated that *A. terricola* used in this work was more tolerant to tea saponin and can be used as feed microorganism for COM fermentation.

### Stress mechanism of maintaining cell homeostasis in *A. terricola* response to tea saponin

As we knew, high concentration of chemicals could cause various damages to cell, and then ultimately affect the reproduction and growth, physiological activity, and even cell death for microorganism (Zhang et al. 2018). In this work, the cell wall and cell membrane in both mycelium and spore are not tightly bound to each other, the boundaries of both cell wall and cell membrane were also irregular and blurred, the contents in vacuole leak out, and the structure of internal organelle was significantly damaged (Figs. 2 and 3). These results indicated that tea saponin (10 mg/mL) changed the normal morphology of mycelia and spore, and then caused an adverse effect on growth and physiological activity of *A. terricola*, which was consistent with previous reported that tea saponin from COM could destroy the cell membrane structure, reduced cell adhesion and aggregation of *Candida albicans*(Yu et al. 2022).

CFW is a kind of fluorescent solution that can stain chitin, which is the main component in fungal cell wall, and its fluorescence value can reflect the integrity of cell wall and the level of chitin content (Riccardi and Nicoletti 2006). In this work, difference in CFW fluorescence value indicated that cell wall integrity of *A. terricola* was not affected by tea saponin (Fig. 4a) and the regulation of cell wall integrity may be attributed to the promotion of chitin biosynthesis in *A. terricola* (Fig. 7c), which in turn protected cell growth and reproduction.

As we knew, tea saponin displays amphiphilic behavior when it interacts with the cell membrane, allowing its polar hydroxyl group to align with the aqueous phase through hydrogen bonding and its nonpolar carbon chain with the lipid phase through dispersion forces, altering cell membrane fluidity, and ultimately disrupting the cellular integrity (Yu et al. 2022). PI as a cell membrane fluorescent dye can not penetrate intact biological membranes. When the cell membrane is damaged, PI can enter the cell and bind DNA to produce fluorescence, which can reflect the integrity of cytomembrane (Helander and Mattila-Sandholm 2000). In this work, the decrease in PI value indicated that the integrity of cytomembrane of *A. terricola* was decreased, which caused an untightly fitting between cell wall and cell membrane(Figs. 2c and 3b and c). The difference in PI value for tea saponin addition (1.25 mg/mL ~ 10 mg/mL) was not significant and might be attributed to the promotion of ergosterol biosynthesis(Fig. 7b), and also further indicated that 1.25 mg/mL and 10 mg/mL may be the MIC and MBC for *A. terricola* against tea saponin, respectively.

The fluorescence intensity of NPN, as a hydrophobic fluorescent probe, is high in not aqueous but hydrophobic environment, and which can be used to characterize the permeability of plasma membrane (Lin et al. 2016). The results about a decrease in NPN fluorescence value indicated the cell membrane structure of *A. terricola* was destroyed by tea saponin and the hydrophobic environment of cell membrane could not be maintained (Fig. 4c). Meantime, tea saponin (2.5 mg/mL to 5 mg/mL) induced cell to produce relative protection mechanisms for reducing the cell membrane permeability and thus maintain the membrane homeostasis in *A. terricola* (Fig. 7b).

When the cytomembrane has been destroyed, the internal electrolyte and electrical conductivity in cell will leak out and increase. The relative electrical conductivity of cell can reflect the permeability of cytomembrane (Bi et al. 2021). In this work, the increase in the relative electrical conductivity indicated that tea saponin addition concentration was positively proportional to cell permeability of *A. terricola* (Fig. 4d). Meantime, tea saponin addition (2.5 mg/mL ~ 5 mg/mL) induced cell to produce relative protection mechanisms for reducing cell membrane permeability and balancing conductivity of cell between intracellular and extracellular, and thus maintain the membrane homeostasis in *A. terricola* (Fig. 4d). In this work, the balance of cell membrane homeostasis in *A. terricola* tolerance to tea saponin may be attributed to the promotion of ergosterol biosynthesis (Fig. 7b).

### Stress mechanism of enzymes protecting system in *A. terricola* response to tea saponin

As we knew, in order to survive under high concentrations of chemicals and ensure the growth and reproduction of microorganisms as normal as possible, fungus uses multiple protection mechanisms to protect and adapt itself, such as photoreactivation and nucleotide excision repair (NER) to deal with DNA damage, heat shock protein (Hsp) to maintain the correct conformation of the protein and assist in the degradation of misfolded or even aggregated proteins, and so on (Zhang et al. 2018). CAT, POD, SOD and GSH-Px, as the important antioxidant enzymes, are widely present in the body and protect cells and tissues from interference and damage by oxides (Ismail et al. 2022). CAT, the marker enzyme of peroxisome, can catalyze the decomposition of hydrogen peroxide into oxygen and water. POD, which can use hydrogen peroxide as the electron acceptor to catalyze the substrate oxidation, has the dual effect to eliminate the toxicity of hydrogen peroxide and phenols, amines, aldehydes and benzene. GSH-Px can reduce toxic peroxides into non-toxic hydroxyl compounds(Missall et al. 2004). SOD, as a natural superoxide-free radical scavenger, can convert harmful superoxide free radicals into hydrogen peroxide and decomposes them into water by CAT and POD. SOD, CAT and POD can form a complete antioxidant chain to resist and block the damage caused by oxygen free radicals to cells and repair damaged cells in time. In this work, the increase of ROS level caused by tea saponin indicated oxidative stress was generated in *A. terricola* (Fig. 5a). The activity decrease in CAT (response to oxidative stress) (Fig. 5e), SOD (destroys radicals) (Fig. 5d) and GSH-PX (Fig. 5c), and increase in POD (Fig. 5b) indicated that not SOD, CAT and GSH-PX but POD did play a role in reducing or eliminating ROS, and then protected or repaired the damage caused by ROS(Zhao et al. 2015).

### Stress mechanism to balance energy metabolism in *A. terricola* response to tea saponin

As we knew, when the cytochrome respiratory chain pathway was inhibited, the alternative respiratory pathway (including the NADH and alternative oxidase (AOX) will be activated to balance material and energy metabolism, which directly transfers electrons from NADH to molecular oxygen to form water without passing through complex III and IV but ubiquinone (Li et al. 2011). In present work, the activity of complex I, II, III and IV all have been inhibited by tea saponin (Fig. 6b) and indicated cytochrome respiratory pathway in *A. terricola* was disrupted. The energy release and ATP generation (Fig. 6a) in *A. terricola* may be achieved through the increase in activity of not Ca^2+^/Mg^2+^-ATPase but Na^+^/K^+^-ATPase (Fig. 6c). These results may also indicated that the conductivity equilibrium in *A. terricola* may be realized as a Na^+^/K^+^ equilibrium (Fig. 6c).

### COM significantly promoting the biosynthesis of cordycepic acid and ergosterol from *A. terricola*

The production of COM is about > 690,000 metric tonnes each year in China (Luan et al. 2020). Nowadays, COM was mainly used as fertilizer or discarded after tea saponin extraction (Yu and He 2018; Luan et al. 2020). However, other nutrients such as protein and polysaccharide in COM were not fully utilized, which was a great waste of *C. oleifera* resources (Luan et al. 2020). Therefore, it is still necessary to explore a preferred technique to efficient utilize the COM with full-resource and high-value. Because of the imbalance of nutrition (especially protein) and the presence of a variety of anti-nutritional factors (saponin, tannin, caffeine, etc.), COM can only be used as feed additives to partially replace RB, WB and soybean meal. ATCs, as dietary supplementation in animal feed, can improve growth performance and immune function of animal, which was ascribe to the abundance of bioactive substances in *A. terricola* (Wang et al. 2019, 2023), including cordycepin, cordycepic acid, D-mannose, and so on.

As showed in above results, low concentration of tea saponin (≤ 5 mg/mL) could significantly promote cordycepic acid production by *A. terricola* (Fig. 7a), and high concentration of tea saponin (≥ 10 mg/mL) could significantly inhibit the growth and physiological activity of *A. terricola* (Figs. 1, 2 and 3). Therefore, the content of tea saponin in COM has been decreased from 21.2% (mg/100 g) to 2.6% (mg/100 g) by microwave-assisted extraction (He et al. 2014) in order to benefit for *A. terricola* growing and promoting cordyceps acid production.

In present work, WB and RB could all promote production not only cordycepic acid (Fig. 8a) but also ergosterol (Fig. 8b) compared to COM. Interestingly, combination (COM: RB: WB = 15:65:20) was more favorable to promote the production of cordycepic acid (Fig. 8a) and ergosterol (Fig. 8b) compared to using RB, WB and COM alone. Meantime, compared to RB and WB, the insignificant difference in content of sugar (Fig. 8c and d) and protein (Fig. 8e and f) in ATCs using combination (COM: RB: WB = 15:65:20) solid fermentation indicated it is feasible to partially substitute WB and RB using desaponified COM to prepare ATCs, which met the nutrient requirements of poultry (National Research Council (U.S.) 1994).

## Conclusions

In this study, the MIC and MBC of tea saponin on *A. terricola* have firstly been determined, then the tolerance concentration of tea saponin, which could significantly promote production of cordyceps acid and ergosterol, has been found based on the stress-protective mechanism of *A. terricola* tolerance to tea saponin. At last, it was demonstrated that COM could partially replace RB and or WB to prepare ATCs through *A. terricola* for the production of antibiotic-free feed additives.

## Data Availability

There are no restrictions on materials and data availability. in addition, some or all data, models and materials generated or used during the study are available from the corresponding authors upon request. Finally, all methods were carried out in accordance with relevant guidelines and regulation.
